# Combined respiratory muscle training and photobiomodulation improve systemic and tissue-specific redox homeostasis in a rat model of type 2 diabetes

**DOI:** 10.1007/s00592-026-02723-3

**Published:** 2026-06-25

**Authors:** Maria Elaine Trevisan, Nubia Gonzatti, Carlos Cassiano Figueiró da Silva, Larissa da Silva Tonetto, Camille Gaube Guex, Gustavo Orione Puntel, Antônio Marcos Vargas da Silva, Luis Ulisses Signori, Cinthia Melazzo de Andrade, Pedro Dal Lago, Liliane de Freitas Bauermann, Rodrigo Boemo Jaenisch

**Affiliations:** 1 Department of Physiotherapy and Rehabilitation, Universidade Federal de Santa Maria, Santa Maria, RS Brazil; 2 Postgraduate Program in Movement Sciences and Rehabilitation, Universidade Federal de Santa Maria, Santa Maria, RS Brazil; 3 Pharmacology Postgraduate Program, Universidade Federal de Santa Maria, Santa Maria, RS Brazil; 4 Departament of Morphology, Universidade Federal de Santa Maria, Santa Maria, Brazil; 5 Department of Small Animal Clinic, Center of Rural Sciences, Universidade Federal de Santa Maria, Santa Maria, RS Brazil; 6 Physical Therapy Departament, Universidade Federal de Ciências da Saúde de Porto Alegre, Porto Alegre, RS Brazil

**Keywords:** Respiratory muscle training, Photobiomodulation, Diabetes, Oxidative stress, Redox homeostasis

## Abstract

**Aims:**

Oxidative stress plays a central role in the pathophysiology of type 2 diabetes and contributes to multiorgan dysfunction. Non-pharmacological strategies targeting redox imbalance may provide complementary benefits beyond glycemic control. This study investigated the effects of combined respiratory muscle training (RMT) and photobiomodulation (PBM) on systemic and tissue-specific redox homeostasis in an experimental model of type 2 diabetes.

**Methods:**

Male rats were allocated into four groups: sedentary Sham (non-diabetic), Sham submitted to RMT combined with PBM, sedentary type 2 diabetes, and type 2 diabetes submitted to the RMT/PBM protocol. Type 2 diabetes was induced by a high-fat diet associated with low-dose streptozotocin. Interventions were performed five days per week for six weeks. Hematological and biochemical parameters were evaluated. Oxidative stress was assessed by lipid peroxidation (thiobarbituric acid reactive substances) and reactive oxygen species production (dichlorofluorescein), while antioxidant defenses were determined by superoxide dismutase activity and non-protein thiol levels in plasma and multiple tissues.

**Results:**

In diabetic rats, the combined RMT/PBM protocol increased mean corpuscular hemoglobin concentration, reduced leukocyte and monocyte counts, and restored plasma protein levels. Oxidative damage was attenuated in plasma, diaphragm, lungs, and kidneys, while antioxidant defenses were enhanced, particularly in the diaphragm, heart, and kidneys. In normoglycemic rats, RMT/PBM also enhanced tissue antioxidant capacity, indicating preserved redox responsiveness.

**Conclusions:**

Combined RMT and PBM promote systemic and tissue-specific redox adaptations and improve hematological and biochemical parameters in diabetic rats. These findings support the potential of this combined intervention as a non-pharmacological strategy to mitigate oxidative dysfunction associated with type 2 diabetes.

## Introduction

Type 2 diabetes (T2D) is a progressive metabolic disorder characterized by chronic hyperglycemia resulting from the combined effects of insulin resistance and β-cell dysfunction, leading to multisystem metabolic impairment [1, 2]. Beyond glycemic dysregulation, T2D is increasingly recognized as a systemic condition marked by metabolic inflexibility, chronic low-grade inflammation, accelerated biological aging, and diabetic sarcopenia [3–5]. These alterations contribute to impaired function across cardiovascular, renal, neural, and musculoskeletal tissues [6, 7].

Oxidative stress represents a central and unifying mechanism underlying many diabetes-related complications. Chronic hyperglycemia exacerbates reactive oxygen species generation via mitochondrial electron transport chain overload, advanced glycation end-product formation, and NADPH oxidase activation, ultimately promoting redox imbalance [4, 5]. Persistent redox imbalance contributes to insulin resistance, endothelial dysfunction, tissue inflammation, and progressive organ damage [5, 7]. Importantly, oxidative injury may persist despite adequate glycemic control, reinforcing the need for therapeutic strategies that directly target redox pathways rather than glucose lowering alone [5].

Skeletal muscle dysfunction is a hallmark of T2D and is closely linked to oxidative damage and chronic inflammation, ultimately contributing to sarcopenia and reduced functional capacity [6]. Among skeletal muscles, the diaphragm and other respiratory muscles appear particularly vulnerable to oxidative damage induced by hyperglycemia. Clinical evidence demonstrates that individuals with diabetes exhibit reduced lung function, impaired strength and endurance of respiratory muscles, and decreased exercise tolerance [8, 9]. In experimental models, diaphragm weakness has been directly linked to oxidative stress–mediated impairments in excitation–contraction coupling and fatigue resistance [10, 11]. Respiratory muscle dysfunction may further exacerbate systemic limitations via activation of the respiratory muscle metaboreflex, redistributing blood flow away from locomotor muscles and increasing sympathetic vasoconstrictor activity [12, 13]. In patients with T2D, this exaggerated reflex contributes to impaired skeletal muscle perfusion and reduced exercise tolerance [14], often limiting adherence to conventional aerobic exercise despite its established metabolic benefits [15].

In this context, respiratory muscle training (RMT) has emerged as a low-burden intervention capable of inducing systemic adaptations that extend beyond the respiratory system. Systematic reviews have shown that RMT improves inspiratory muscle strength, autonomic modulation, and functional capacity in patients with diabetes [16, 17]. Preclinical studies further indicate that RMT enhances diaphragmatic oxidative enzyme activity, improves hemodynamic and autonomic control, and attenuates oxidative DNA and lipid damage [18–21].

Photobiomodulation (PBM) represents another non-invasive strategy with well-established effects on inflammation and redox homeostasis. PBM primarily acts via cytochrome c oxidase activation, enhancing mitochondrial efficiency, nitric oxide signaling, and antioxidant defenses [22, 23]. Clinical trials in individuals with T2D have reported improvements in hemodynamic responses and redox balance following PBM [23, 24], while experimental studies consistently demonstrate reductions in lipid peroxidation and upregulation of antioxidant enzymes in skeletal muscle, heart, and kidneys [25, 26].

Although RMT and PBM have been associated with modulation of redox balance and inflammatory processes in diabetic conditions, their combined effects remain largely unexplored. Given that RMT provides a targeted mechanical and metabolic stimulus [19, 20], while PBM may exert photochemical effects at the cellular level [22, 23], their integration may promote complementary biological responses across tissues affected by type 2 diabetes. Therefore, the present study aimed to investigate the effects of a combined RMT and PBM protocol on systemic and tissue-specific redox markers, as well as hematological and biochemical parameters, in a rat model of type 2 diabetes induced by a high-fat diet and low-dose streptozotocin. We hypothesized that the combined RMT/PBM intervention would improve oxidative balance and systemic biological parameters independently of glycemic normalization.

## Methods

### Animals

This study was approved by the Institutional Committee for Animal Use (CEUA) of the Universidade Federal de Santa Maria (UFSM) under protocol number 9,241,020,620. All experimental procedures were conducted in accordance with the Principles of Laboratory Animal Care (NIH publication No. 86 − 23, revised 1985) and in compliance with applicable national legislation for the care and use of laboratory animals. Male Wistar rats (200–260 g) were housed under controlled conditions (21 °C, 50–60% humidity, 12-h light/dark cycle; three per cage) with free access to food and water.

### Induction of type 2 diabetes

T2D was induced using a hybrid protocol combining a hight-fat diet and low-dose streptozotocin. Rats assigned to the T2D group received high-fat diet (70% standard chow, 15% sucrose, 10% fat, 5% egg yolk) for four weeks, while Sham rats remained on a standard diet. Following a 12-h fast, T2D rats received a single intraperitoneal injection of streptozotocin (35 mg/kg, citrate buffer, pH 4.5), whereas Sham animals received the vehicle. One week later, animals with fasting blood glucose > 200 mg/dL were considered diabetic [18, 25, 27]. Animals not meeting this criterion were excluded.

### Experimental design

After diabetes confirmation, rats were randomly assigned to four groups: Sedentary-Sham (Sed-Sham), RMT/PBM-Sham, Sedentary-T2D (Sed-T2D), and RMT/PBM-T2D. The intervention was performed 5 days per week for 6 weeks. To avoid acute physiological overlap, RMT and PBM were conducted in separate daily sessions.

### RMT/PBM protocol

RMT was performed using a customized whole-body acrylic chamber with inspiratory resistive loading, as previously described [18, 21, 28, 29]. After a 5-day familiarization period, inspiratory resistance was progressively increased over the first two weeks by reducing the orifice diameter (0.8–0.3 mm) [18, 19, 21]. PBM was delivered using a continuous-wave diode laser (InGaAlP, 660 nm, 20 mW; ENDOPHOTON-LLT-0107, KLD Biosystems). A daily dose of 21 J/cm² was applied to two points over the right gastrocnemius muscle (spot size 0.035 cm²; 36.75 s per point), with the probe positioned perpendicular to the skin, as previously described [25, 30]. Twenty-four hours after the final session, rats were anesthetized with isoflurane (4%), and blood was collected by cardiac puncture [18, 25]. The diaphragm, gastrocnemius, lungs, heart, kidneys, and liver were excised, weighed, flash-frozen, and stored at –80 °C for subsequent analyses.

### Hematological and biochemical analysis

Blood samples were processed for hematological and biochemical analyses using automated analyzers. Hematological parameters included red blood cells, hemoglobin concentration, mean corpuscular volume, mean corpuscular hemoglobin concentration, white blood cells, neutrophils, lymphocytes, monocytes, and platelets, and were determined using an automated hematology analyzer (Mindray^®^ BC-2800Vet). Serum biochemical analyses were performed using an automated biochemical analyzer (BioClin^®^ 2000 Plus, MG, Brazil) and manufacturer-validated reagents (BioClin^®^). The biochemical panel included albumin, creatinine, and urea [31].

### Oxidative stress and antioxidant defenses

Plasma and tissue homogenates were prepared as previously described [32]. Protein concentration was determined using the Lowry method [33].

*Thiobarbituric Acid Reactive Substances (TBARS)*. Lipid peroxidation was assessed by TBARS according to the method of Ohkawa et al. [34]. A total of 40 µL of plasma was incubated with 20 µL of distilled water, 100 µL of acetic acid, 100 µL of thiobarbituric acid, and 40 µL of sodium dodecyl sulfate, followed by heating at 100 °C for 120 min. TBARS concentrations were calculated from a malondialdehyde standard curve and normalized to total protein content [34].

*Dichlorofluorescein (DCF)*. Reactive species formation was assessed using dichlorodihydrofluorescein diacetate (DCFH-DA), following a modified protocol of Perez-Severiano et al. [35]. Plasma and tissue homogenates (50 µL) were incubated in 10 mM Tris-HCl buffer (pH 7.4) containing 1 µM DCFH-DA for 60 min in the dark. DCF-derived fluorescence was quantified using a DCF standard curve and expressed relative to protein content [35].

*Superoxide Dismutase (SOD)*. SOD activity was determined spectrophotometrically according to Misra and Fridovich [36]. Homogenized samples of plasma, muscle, and organs were added to a reaction medium containing 2 mM EDTA in 50 mM NaHCO₃/Na₂CO₃ buffer (pH 10.3). Enzymatic activity was initiated by the addition of 4 mM epinephrine, and the rate of epinephrine autoxidation was monitored. SOD activity was expressed as units of enzymatic activity per milligram of protein [36].

*Non-protein thiols (NPSH)*. NPSH levels were obtained by mixing 134 µL of sample with 67 µL of 5% trichloroacetic acid. The supernatant (60 µL) was added to a reaction medium containing potassium phosphate buffer (pH 7.4), distilled water, and DTNB. NPSH levels were calculated from a glutathione calibration curve and normalized to protein content [37].

### Statistical analysis

Data are presented as mean ± standard deviation. Normality was assessed using the Shapiro–Wilk test. Two-way ANOVA was applied to examine the effects of diabetes (T2D vs. Sham) and intervention (RMT/PBM vs. Sedentary), followed by Tukey’s post hoc test. Effect sizes were calculated using Hedges’s g and interpreted as trivial (< 0.2), small (0.2–0.49), moderate (0.5–0.79), or large (≥ 0.8). The significance level of *P* < 0.05 was considered. Analyses were performed using GraphPad Prism 8 (GraphPad Software, San Diego, CA, USA). Due to the nature of tissue collection and biochemical assays, minor variations in sample size were observed across experimental groups. These differences resulted from occasional sample loss during tissue harvesting and processing, as well as insufficient sample volume for specific analyses. The exact sample size for each variable is indicated in the corresponding figures.

## Results

### Metabolic and morphologic parameters

**Fig. 1:**
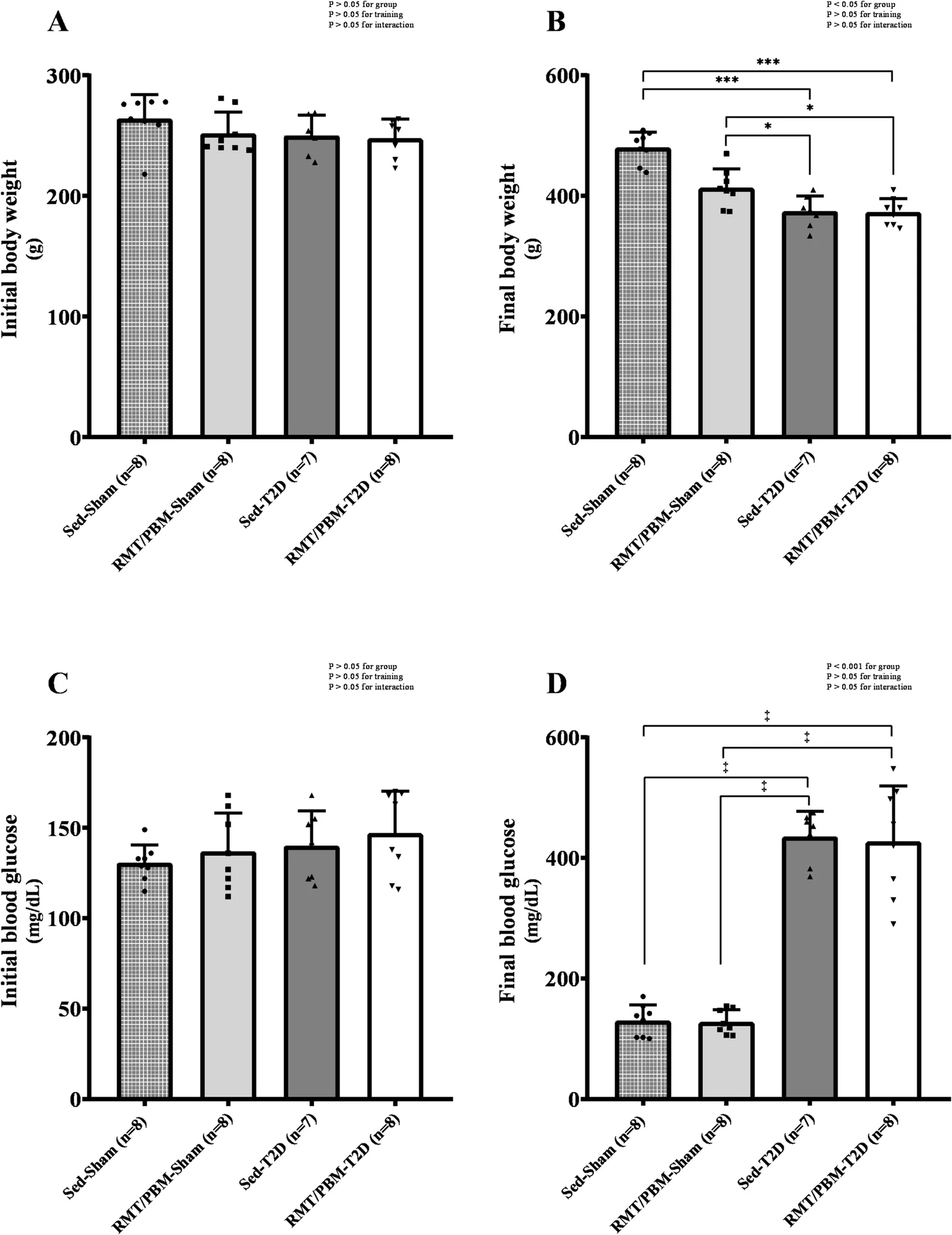
Values are means ± SD. *P* < 0.05. The groups were compared by two-way ANOVA and Tukey post hoc tests. Sedentary-Sham (Sed-Sham), RMT/PBM-Sham, Sedentary-T2D (Sed-T2D) and RMT/PBM-T2D. *P < 0.05 compared with Sed-T2D and RMT/PBM-T2D; ***P < 0.001 compared with Sed-T2D and RMT/PBM-T2D; ‡*P* < 0.0001 compared with Sed-Sham and RMT/PBM-Sham

Thirty-two animals began the study. One rat in the Sed-T2D group died, resulting in a final sample size of *n* = 31. Final body weight was lower in T2D compared to Sham rats (*P* < 0.05 for group effect). Fasting blood glucose was higher in T2D compared to Sham groups (> 200 mg/dL; *P* < 0.001 for group effect). The 6‑week RMT/PBM protocol did not change fasting glucose or final body weight. Data are summarized in Fig. 1. Heart, diaphragm, and gastrocnemius weights did not differ between groups.

### Hematological and biochemical profile

In diabetic rats, RMT/PBM reduced white blood cell count (*P* < 0.05 for group and interaction effects) with a large effect size (g = − 1.73), and monocytes (*P* < 0.01 for group and training effects, and *P* < 0.05 for interaction effect), with a large effect size (g = − 2.31), compared to Sed-T2D rats. RMT/PBM also increased mean corpuscular hemoglobin concentration in T2D rats compared to Sham rats (*P* < 0.001 for group effect). In Sham rats, RMT/PBM increased mean corpuscular volume compared to Sed-Sham and Sed-T2D rats (*P* < 0.05 for the training effect), with a large effect size (g = 1.41). No differences in platelets, neutrophils, lymphocytes, red blood cell counts, or hemoglobin concentration levels were observed between groups. Total plasma protein was elevated in Sed-T2D compared to Sham rats (*P* < 0.05 for group effect). RMT/PBM intervention reduced plasma protein levels in T2D rats compared to Sed-T2D (*P* < 0.05 for group effect). No differences in albumin, creatinine, or urea levels were observed between groups. These data are summarized in Table 1.

**Table 1:** Hematological and biochemical parameters of the experimental groups

Parameters	Sed-Sham	RMT/PBM-Sham	Sed-T2D	RMT/PBM-T2D
Hematological parameters				
Red blood cells (x10⁶/µL)	8.43 ± 0.43	8.23 ± 0.50	8.50 ± 0.40	7.97 ± 0.26
Hemoglobin (g/dL)	16.54 ± 0.80	16.98 ± 0.68	17.37 ± 1.01	17.38 ± 1.37
Mean corpuscular volume (fL)	53.55 ± 1.35	56.21 ± 2.29^a,b^	53.70 ± 0.80	54.15 ± 1.11
Mean corpuscular hemoglobin concentration (g/dL)	36.60 ± 0.89	36.77 ± 0.83	37.89 ± 1.02	40.38 ± 3.64^c^
Platelets (x10³/µL)	867 ± 98	849 ± 97	1096 ± 328	1149 ± 230
White blood cells (x10³/µL)	6400 ± 1067	6300 ± 1152	7071 ± 1145	4900 ± 1352^a^
Neutrophils (x10³/µL)	1282 ± 600	740 ± 406	1447 ± 617	1159 ± 692
Lymphocytes (x10³/µL)	4910 ± 1074	5023 ± 979	4876 ± 1002	3609 ± 716
Monocytes (x10³/µL)	123 ± 103^d^	98 ± 38^d^	302 ± 50	121 ± 98^d^
Biochemical parameters				
Total Protein (g/dL)	6.50 ± 0.30^a^	6.56 ± 0.60^a^	9.95 ± 3.75	7.31 ± 2.61
Albumin (g/dL)	2.89 ± 0.30	2.95 ± 0.22	3.12 ± 0.62	2.70 ± 1.11
Creatinine (mg/dL)	0.63 ± 0.07	0.65 ± 0.05	0.60 ± 0.00	0.67 ± 0.13
Urea (mg/dL)	46.50 ± 6.61	45.87 ± 8.50	53.00 ± 12.52	54.38 ± 10.07

Values are means ± SD. *P* < 0.05. The groups were compared by two-way ANOVA and Tukey post hoc tests. Sedentary-Sham (Sed-Sham, *n* = 8), respiratory muscle training combined PBM (RMT/PBM)-Sham (RMT/PBM-Sham, *n* = 8), sedentary-diabetes (T2D) (Sed-T2D, *n* = 7) and RMT/PBM-T2D (*n* = 6)

^a^*P* < 0.05 comparing with Sed-T2D

^b^*P* < 0.05 comparing with Sed-Sham

^c^*P* < 0.05 comparing with Sed-Sham and RMT/PBM-Sham

^d^*P* < 0.01 comparing with Sed-T2D

### Assessment of oxidative stress markers and antioxidant defenses

#### Oxidative damage markers

**Fig. 2:**
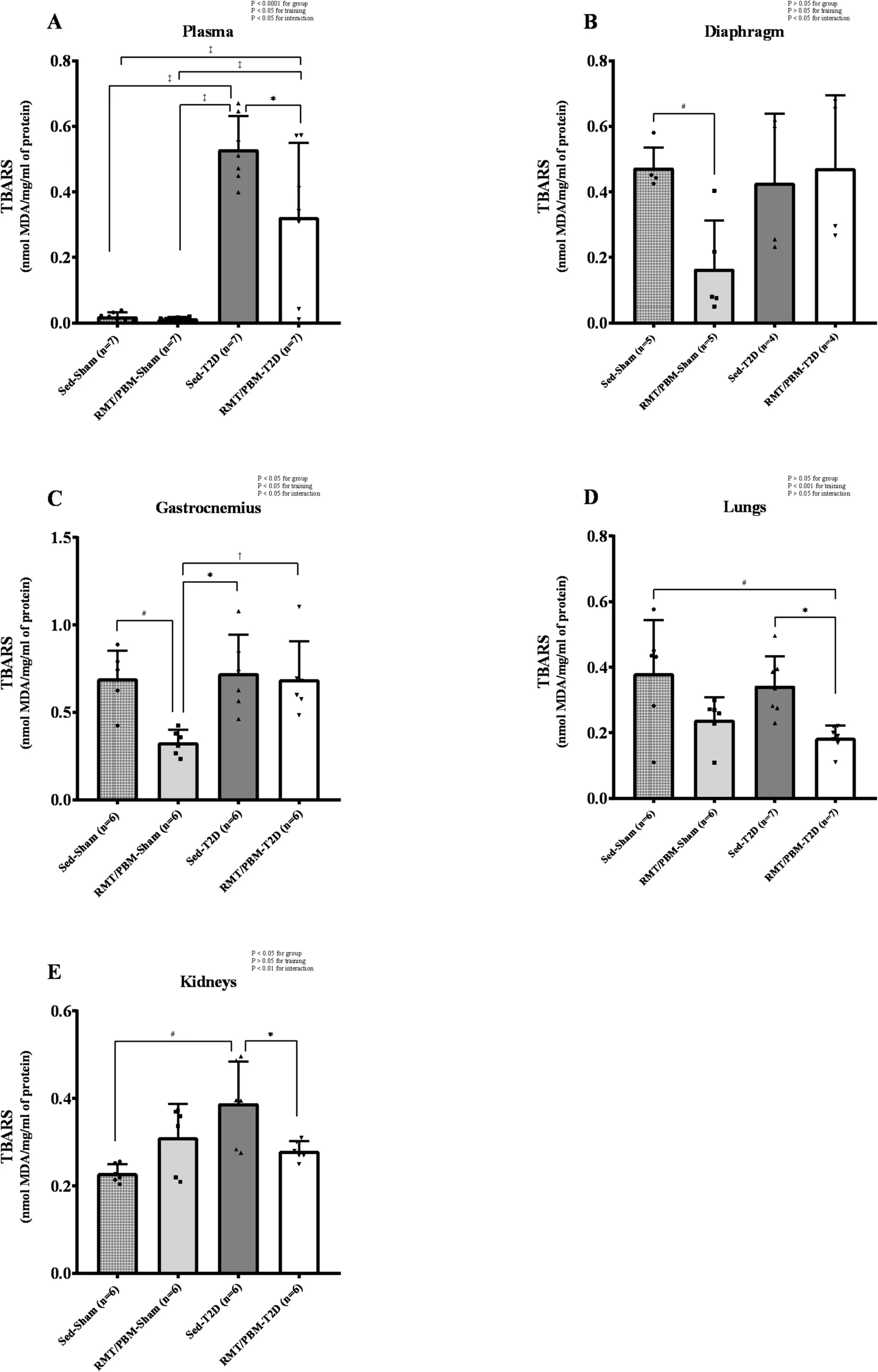
TBARS as a marker of oxidant activity. Values are means ± SD. *P* < 0.05. The groups were compared by two-way ANOVA and Tukey post hoc tests. TBARS: thiobarbituric acid reactive substance levels. ^#^*P* < 0.05 compared with Sed-Sham; *P < 0.05 compared with Sed-T2D; ^†^*P* < 0.05 compared with RMT/PBM-Sham; ^‡^*P* < 0.0001 compared with Sed-Sham and RMT/PBM-Sham

*Thiobarbituric acid reactive substances (TBARS).* Plasma TBARS levels were higher in T2D rats compared to Sed-Sham rats (*P* < 0.0001 for group effect). RMT/PBM reduced plasma TBARS levels in diabetic rats (*P* < 0.0001 for group effect; *P* < 0.05 for training and interaction effects; g = − 1.81) (Fig. 2A). In the lungs, TBARS levels were lower in RMT/PBM-T2D rats compared to Sed-T2D rats (*P* < 0.001 for training effect; g = − 2.29) (Fig. 2D). In the kidneys, TBARS levels were higher in Sed-T2D rats compared to Sed-Sham rats (*P* < 0.05 for group effect) and lower in RMT/PBM-T2D rats compared to Sed-T2D rats (*P* < 0.01 for interaction effect; g = − 1.58) (Fig. 2E). In Sham rats, RMT/PBM decreased TBARS levels in the diaphragm (*P* < 0.05 for interaction effect; g = − 2.72) and in the gastrocnemius (*P* < 0.05 for group, training, and interaction effects; g = − 2.95) compared to Sed-Sham rats (Fig. 2B and C). TBARS levels in the diaphragm and gastrocnemius did not differ between Sed-T2D rats and RMT/PBM-T2D rats.

**Fig. 3:**
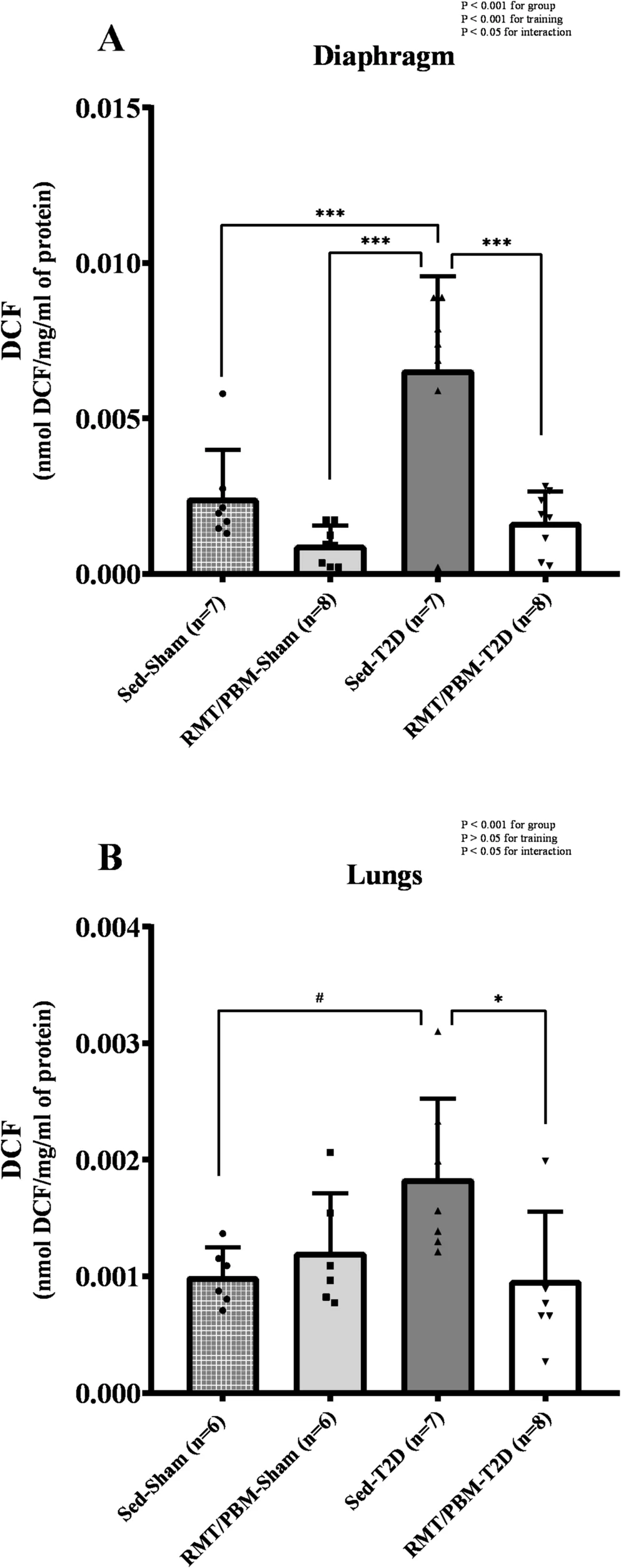
DCF as a marker of oxidant activity. Values are means ± SD. *P* < 0.05. The groups were compared by two-way ANOVA and Tukey post hoc tests. DCF: dichlorofluorescein levels. ^#^*P* < 0.05 compared with Sed-Sham; *P < 0.05 compared with Sed-T2D; ***P < 0.001 compared with Sed-Sham, RMT/PBM-Sham, and RMT/PBM-T2D

*Dichlorofluorescein (DCF) levels.* DCF levels were higher in the diaphragm and lungs of Sed-T2D rats compared to Sed-Sham rats (both *P* < 0.001 for group effect). RMT/PBM reduced DCF levels in the diaphragm of T2D rats (*P* < 0.001 for group and training effects, *P* < 0.05 for interaction effect; g = − 2.19) and in the lungs of T2D rats (*P* < 0.05 for interaction effect; g = − 1.37) compared to Sed-T2D rats (Fig. 3A and B).

#### Antioxidant Defenses

**Fig. 4:**
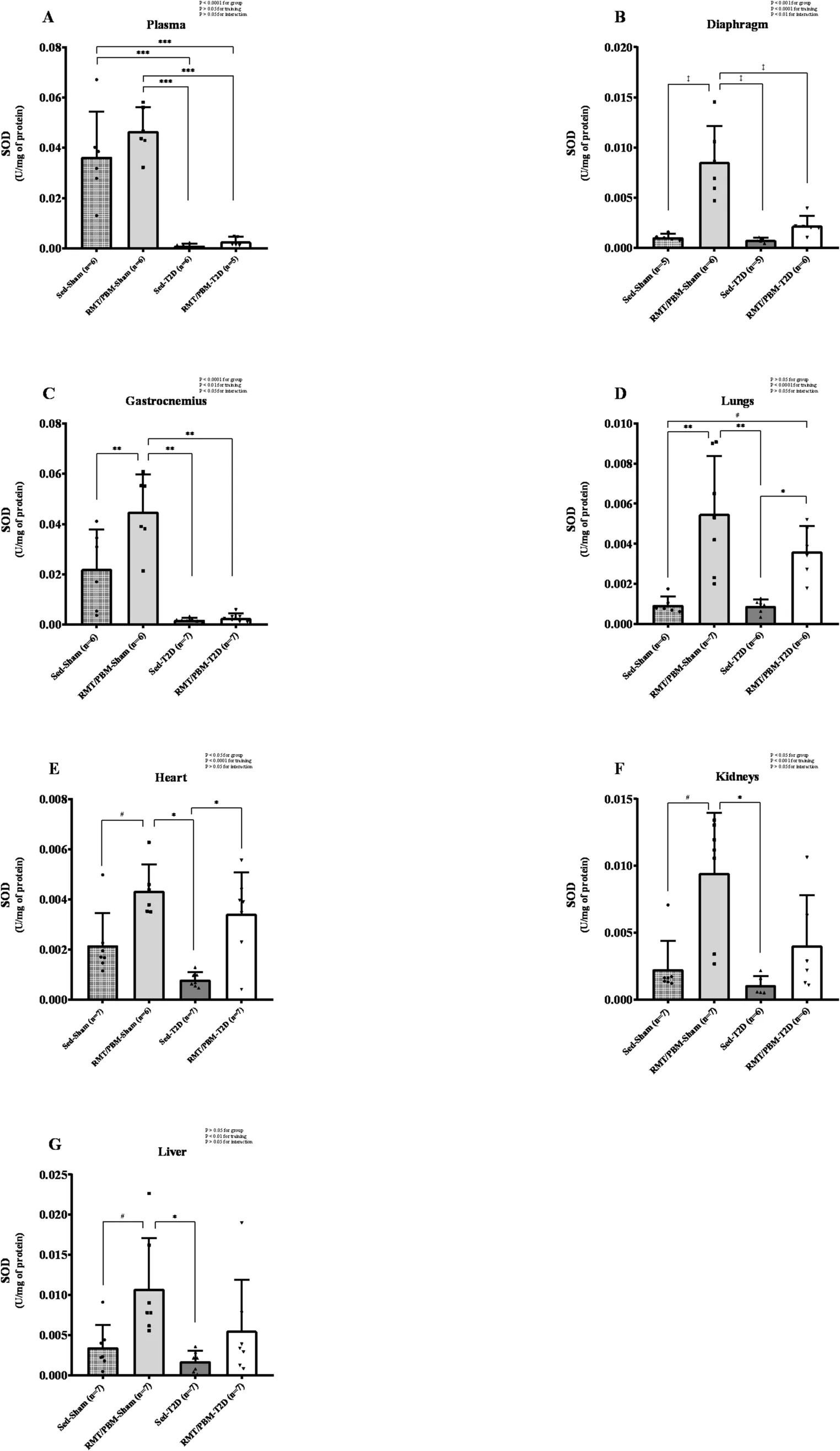
SOD as a marker of antioxidant activity. The groups were compared by two-way ANOVA and Tukey post hoc tests. SOD: superoxide dismutase levels. ^#^*P* < 0.05 compared with Sed-Sham; *P < 0.05 compared with Sed-T2D; **P < 0.01 compared with Sed-Sham, Sed-T2D, and RMT/PBM-T2D; ***P < 0.001 compared with Sed-T2D and RMT/PBM-T2D; ^‡^*P* < 0.0001 compared with Sed-Sham, Sed-T2D, and RMT/PBM-T2D

*Superoxide dismutase (SOD) activity.* Plasma SOD activity was lower in Sed-T2D rats compared to Sed-Sham rats (*P* < 0.0001 for group effect). RMT/PBM did not modify plasma SOD activity in T2D rats (Fig. 4A). In the lungs, RMT/PBM increased SOD activity in both T2D (*P* < 0.0001 for training effect; g = 2.94) and Sham (*P* < 0.0001 for training effect; g = 2.20) rats compared to their respective sedentary controls (Fig. 4D). In the heart, SOD activity was higher in RMT/PBM-T2D (*P* < 0.05 for group effect, *P* < 0.0001 for training effect; g = 2.21) and RMT/PBM-Sham (g = 1.85) rats compared to their sedentary controls (Fig. 4E). In Sham rats, RMT/PBM increased SOD activity in the diaphragm (*P* < 0.001 for group effect, *P* < 0.0001 for training effect, *P* < 0.01 for interaction effect; g = 2.95), gastrocnemius (*P* < 0.0001 for group effect, *P* < 0.01 for training effect, *P* < 0.05 for interaction effect; g = 1.49), kidneys (*P* < 0.05 for group effect, *P* < 0.001 for training effect; g = 2.04), and liver (*P* < 0.01 for training effect; g = 1.49) compared to Sed-Sham rats (Fig. 4B, C, F and G). No significant changes in SOD activity were observed in the gastrocnemius, kidneys, or liver of T2D rats following the RMT/PBM protocol (Fig. 4C, F and G).

**Fig. 5:**
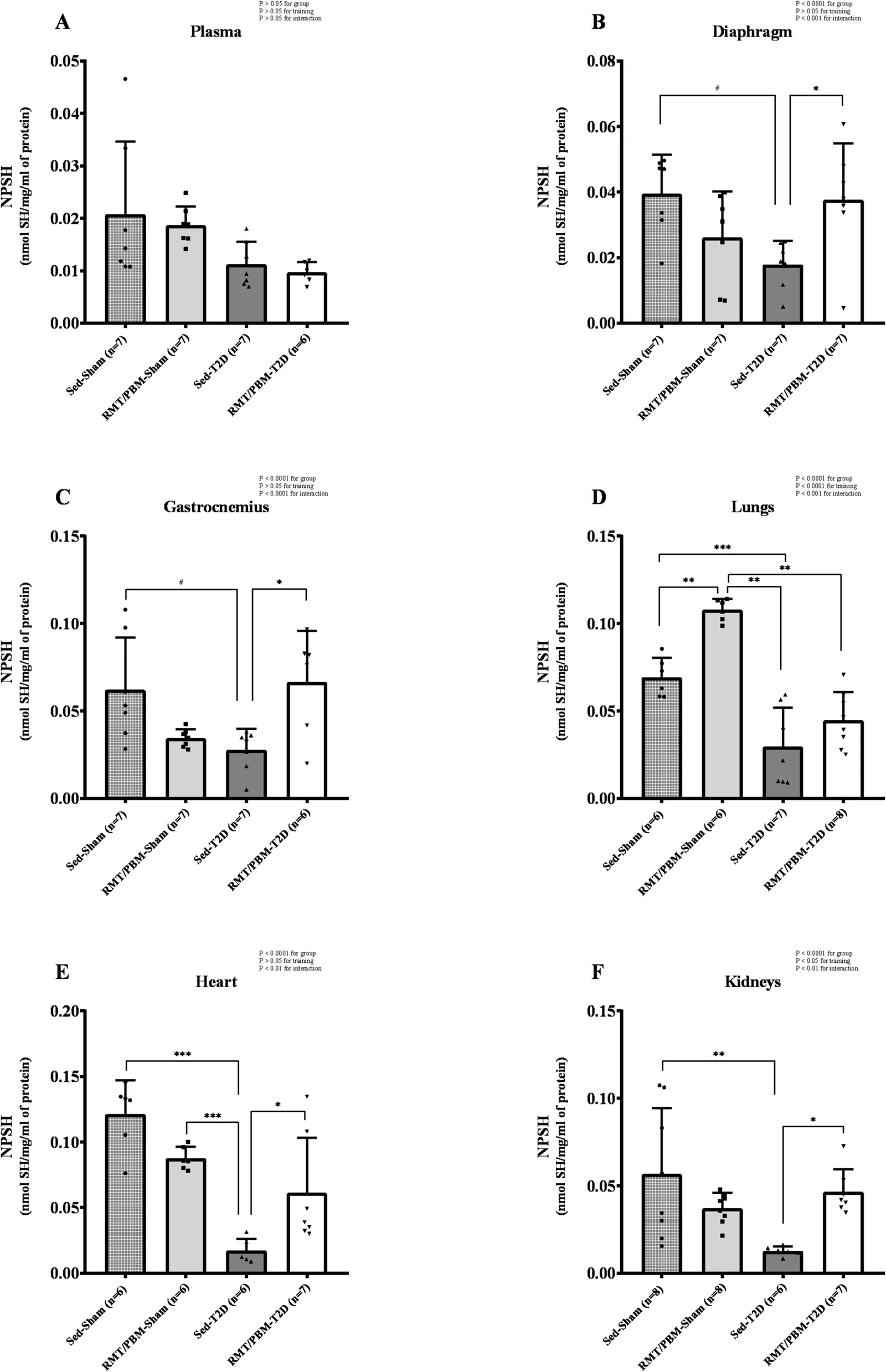
NPSH as a marker of antioxidant activity. The groups were compared by two-way ANOVA and Tukey post hoc tests. NPSH: non-protein thiol levels. ^#^*P* < 0.05 compared with Sed-Sham; *P < 0.05 compared with Sed-T2D; **P < 0.01 compared with Sed-Sham, Sed-T2D, and RMT/PBM-T2D; ***P < 0.001 compared with Sed-T2D.

*Non-protein thiol (NPSH) levels.* Plasma NPSH levels did not differ between groups. In the diaphragm, NPSH levels were lower in Sed-T2D rats compared to Sed-Sham rats (*P* < 0.0001 for group effect) and higher in RMT/PBM-T2D compared to Sed-T2D rats (*P* < 0.001 for interaction effect; g = 1.50) (Fig. 5B). Similarly, in the gastrocnemius, NPSH levels were lower in T2D rats compared to Sham rats (*P* < 0.0001 for group effect) and higher in RMT/PBM-T2D compared to Sed-T2D rats (*P* < 0.0001 for interaction effect; g = 1.74) (Fig. 5C). In the heart, NPSH levels were lower in Sed-T2D rats compared to Sed-Sham rats (*P* < 0.0001 for group effect) and higher in RMT/PBM-T2D rats compared to Sed-T2D rats (*P* < 0.01 for interaction effect; g = 1.44) (Fig. 5E). In the kidneys, NPSH levels were lower in Sed-T2D compared to Sed-Sham rats (*P* < 0.0001 for group effect) and higher in both RMT/PBM-T2D (g = 2.82) and RMT/PBM-Sham rats compared to sedentary rats (*P* < 0.01 for interaction effect) (Fig. 5F). In the lungs, NPSH levels were lower in Sed-T2D rats compared to Sed-Sham rats (*P* < 0.0001 for group effect). RMT/PBM did not alter NPSH levels in rats with type 2 diabetes, but increased them in Sed-Sham (*P* < 0.0001 for group and training effects; g = 4.27) (Fig. 5D).

## Discussion

The present study provides integrated preclinical evidence that a combined RMT and PBM protocol improves systemic and tissue-specific redox homeostasis in a rat model of type 2 diabetes induced by a high-fat diet and low-dose streptozotocin. Notably, the intervention was associated with consistent reductions in oxidative damage markers and enhancement of antioxidant defenses in plasma, diaphragm, lungs, heart, and kidneys. These findings suggest that the observed physiological adaptations are, at least in part, mediated by modulation of redox pathways rather than secondary glycemic normalization.

Consistent with previous reports, diabetic animals showed a reduction in final body weight, a characteristic of this experimental model that reflects metabolic inefficiency associated with sustained hyperglycemia, oxidative stress, and low-grade inflammation [3, 18, 25, 38]. In this context, skeletal muscle alterations in diabetes have been associated with increased production of reactive oxygen species and impairments in cellular bioenergetics [3, 6, 7, 39]. Evidence suggests that oxidative stress in skeletal muscle may precede detectable changes in muscle mass, indicating that early cellular impairments do not necessarily translate into overt atrophy [3, 40]. Moreover, skeletal muscle responses in diabetes appear to be highly model-dependent [39]. In the present study, no differences were observed in gastrocnemius muscle weight between diabetic and control (Sham) groups. Consistent with this finding, experimental studies using streptozotocin-based models have reported variable effects on muscle mass, with some demonstrating preserved muscle weight despite metabolic and oxidative alterations [18, 25, 39]. In addition, current evidence indicates that the development of diabetic sarcopenia is influenced by disease duration and severity and is not a universal feature of all type 2 diabetes models [3, 40]. Therefore, the absence of muscle mass reduction in the present study is consistent with the heterogeneity of skeletal muscle responses in experimental diabetes. Accordingly, the preservation of gastrocnemius weight should be interpreted with caution. While it may be consistent with the overall modulation of oxidative stress and inflammatory markers, the study was not specifically designed to assess muscle atrophy or hypertrophy, and no direct evaluation of muscle structure or function was performed.

Importantly, within this preclinical context, the absence of fasting glucose reduction does not preclude relevant biological adaptations. Previous studies have demonstrated that non-pharmacological interventions, such as physical exercise, may induce beneficial effects in diabetes through mechanisms that are, at least in part, independent of glycemic control, including improvements in redox homeostasis and intracellular signaling pathways [41, 42]. In line with these observations, the present study indicates that the combined RMT/PBM intervention elicited measurable systemic and tissue-specific responses, including attenuation of oxidative damage and enhancement of antioxidant defenses, independently of changes in glycemic levels. Collectively, these findings reinforce the concept that non-glycemic pathways, particularly those related to redox balance and cellular signaling, may contribute to physiological adaptations in experimental models of diabetes. While mitochondrial-related processes may also be involved, such mechanisms were not directly assessed in the present study and should therefore be interpreted with caution.

At the hematological level, diabetes-related oxidative stress compromises erythrocyte membrane integrity, increasing susceptibility to lipid peroxidation and eryptosis [43]. The increase in mean corpuscular hemoglobin concentration observed in diabetic animals following the combined intervention may reflect improved erythrocyte stability under conditions of reduced oxidative damage [43]. In contrast, the increase in mean corpuscular volume observed in normoglycemic animals likely represents an adaptive erythropoietic response under preserved metabolic conditions. Among the systemic findings, the reduction in total leukocyte and monocyte counts in diabetic animals following RMT/PBM is particularly relevant, given the established role of monocytes in chronic low-grade inflammation, endothelial dysfunction, and atherosclerotic processes in diabetes [44]. The concomitant normalization of plasma protein levels further supports a systemic effect on inflammatory and homeostatic regulation [4]. These findings are consistent with previous reports indicating that RMT can attenuate oxidative stress and inflammation [18, 45, 46], while PBM has been shown to modulate cytokine signaling and redox-sensitive pathways [22, 23, 25]. However, the present study does not allow direct identification of the molecular pathways involved, and these mechanisms should be interpreted as plausible but not directly demonstrated.

Conversely, the combined RMT/PBM protocol elicited marked improvements in redox homeostasis, as evidenced by decreases in TBARS and DCF levels in plasma, diaphragm, lungs, and kidneys. In parallel, there was an increase in antioxidant defenses, particularly SOD activity and NPSH levels in the diaphragm, heart, and kidneys. In normoglycemic animals, the intervention similarly enhanced endogenous antioxidant capacity across multiple tissues, suggesting a hormetic enhancement of redox resilience even under physiological conditions [5]. This response is consistent with a hormetic framework, although this interpretation should be considered cautiously, as specific signaling pathways were not directly assessed.

From a translational perspective, clinical evidence supports the relevance of targeting oxidative stress through non-pharmacological strategies in patients with cardiometabolic diseases. Randomized controlled trials and systematic reviews have demonstrated that RMT can improve functional capacity, inspiratory muscle strength, and quality of life in clinical populations, including patients with heart failure and metabolic disorders [16, 17, 47, 48]. In parallel, PBM has been associated with reductions in oxidative stress and inflammation, as well as improvements in muscle performance and recovery, as demonstrated in randomized trials and meta-analyses [23–26, 49–51]. Although these studies have not specifically evaluated combined protocols, their findings suggest that both interventions act on complementary biological pathways relevant to redox homeostasis. In this context, the present preclinical findings provide mechanistic support for the hypothesis that combining RMT and PBM may represent a promising adjunctive strategy to mitigate oxidative stress-related damage in T2D. Nevertheless, it is important to emphasize that these implications remain speculative and hypothesis-generating.

The present findings extend previous observations demonstrating that isolated RMT induces tissue-specific redox and bioenergetic adaptations in diabetic and heart failure models [18, 20], while PBM has been shown to improve oxidative damage in diabetes [23, 25]. Importantly, the combined protocol was associated with a broader multiorgan response compared to those typically reported with single-modality interventions. However, direct comparisons between combined and isolated interventions were not performed in the present study, and therefore conclusions regarding additive or synergistic effects should be interpreted with caution. From a mechanistic perspective, the observed effects may reflect the interaction of complementary physiological stimuli. RMT is known to increase metabolic demand, oxygen flux, and redox signaling, particularly in respiratory and locomotor muscles [12, 18, 52]. In parallel, PBM has been associated with mitochondrial electron transport, nitric oxide bioavailability, and antioxidant gene expression through cytochrome c oxidase–dependent pathways [22]. The combination of these stimuli may contribute to the coordinated attenuation of oxidative stress observed across multiple organs. However, the specific molecular pathways underlying these effects were not directly evaluated and remain to be elucidated.

Collectively, these findings suggest that the combined RMT/PBM intervention may contribute to the modulation of redox homeostasis at a systemic level. By targeting processes associated with oxidative stress and inflammation, this approach may have relevance within the context of diabetes-related pathophysiology. Nevertheless, the interpretation of these results should consider several limitations. First, the study design did not include groups receiving isolated interventions, which limits the ability to fully distinguish the independent and potential additive effects of RMT and PBM. However, the combined RMT/PBM protocol was designed to reflect a more clinically relevant multimodal approach, considering that exercise-based strategies represent a cornerstone in the management of type 2 diabetes. Second, although consistent changes in oxidative stress and inflammatory markers were observed across multiple tissues, the absence of direct assessments of mitochondrial function and redox-sensitive signaling pathways limits mechanistic interpretation. Third, functional outcomes, such as exercise capacity, vascular function, or detailed metabolic profiling, were not evaluated, which restricts the translational relevance of the findings. Finally, as a preclinical study, caution is warranted when extrapolating these results to human populations. Further investigations are needed to establish optimal intervention parameters, assess safety, and determine the clinical applicability of this combined approach.

## Conclusions

This study demonstrates that the combined RMT/PBM intervention induces multiorgan responses in an experimental model of type 2 diabetes, characterized by the attenuation of oxidative stress and the modulation of antioxidant defenses. The protocol was associated with reductions in markers of lipid peroxidation and reactive species formation, along with the enhancement of endogenous antioxidant systems in metabolically relevant tissues, including the diaphragm, heart, lungs, and kidneys. Notably, these effects occurred in the absence of changes in glycemic control, indicating that the observed adaptations are primarily related to redox-sensitive pathways. In addition to tissue-specific effects, the intervention was associated with systemic biological responses, including the modulation of circulating inflammatory-related parameters, reinforcing its impact on redox and homeostatic processes. While the underlying mechanisms were not directly assessed, the present findings support the biological relevance of combined non-pharmacological strategies targeting oxidative stress in experimental diabetes. Further studies are warranted to elucidate the mechanisms involved and to determine the potential clinical applicability of this approach.

## Data Availability

All data presented in the present study are available from the corresponding author on reasonable request.

## Abbreviations

DCF: Dichlorofluorescein
NPSH: Non-protein sulfhydryl groups
PBM: Photobiomodulation
RMT: Respiratory muscle training
SOD: Superoxide dismutase
TBARS: Thiobarbituric acid reactive substances
T2D: Type 2 diabetes
